# The effect of age-related hearing loss and listening effort on resting state connectivity

**DOI:** 10.1038/s41598-019-38816-z

**Published:** 2019-02-20

**Authors:** Stephanie Rosemann, Christiane M. Thiel

**Affiliations:** 1 Biological Psychology, Department of Psychology, School of Medicine and Health Sciences, Carl-von-Ossietzky Universität Oldenburg, Oldenburg, Germany; 2 Cluster of Excellence “Hearing4all”, Carl von Ossietzky Universität Oldenburg, Oldenburg, Germany

## Abstract

Age-related hearing loss is associated with a decrease in hearing abilities for high frequencies. This increases not only the difficulty to understand speech but also the experienced listening effort. Task based neuroimaging studies in normal-hearing and hearing-impaired participants show an increased frontal activation during effortful speech perception in the hearing-impaired. Whether the increased effort in everyday listening in hearing-impaired even impacts functional brain connectivity at rest is unknown. Nineteen normal-hearing and nineteen hearing-impaired participants with mild to moderate hearing loss participated in the study. Hearing abilities, listening effort and resting state functional connectivity were assessed. Our results indicate no differences in functional connectivity between hearing-impaired and normal-hearing participants. Increased listening effort, however, was related to significantly decreased functional connectivity between the dorsal attention network and the precuneus and superior parietal lobule as well as between the auditory and the inferior frontal cortex. We conclude that already mild to moderate age-related hearing loss can impact resting state functional connectivity. It is however not the hearing loss itself but the individually perceived listening effort that relates to functional connectivity changes.

## Introduction

Age-related hearing loss–also known as presbyacusis–is one of the most common disorders affecting older adults. Presbyacusis affects primarily high frequencies making speech understanding difficult^1,2^. The degraded auditory input leads to an increased effort to process and understand speech. The term listening effort refers to the process of hearing with intention and attention and therefore involves the allocation of attentional as well as cognitive resources to meet the required cognitive demands^3–5^. Hearing loss hence decreases available resources in charge of cognitive control, because the neural capacities needed for understanding speech in adverse listening conditions decrease the resources available for other cognitive operations^1,6–8^. As several cognitive abilities such as working memory, episodic memory, attention switching and interference control decline with increasing age, these become even more affected during effortful listening^1,9–13^. Moreover, there is evidence that hearing loss impairs long-term memory^14^.

Previous task-based neuroimaging studies show that recruitment of frontal areas is related to increases in listening effort in younger and elderly volunteers to compensate for the decreased auditory input^15–25^. Further, effortful speech perception is associated with an increase in neural activation in areas including the cinguloopercular network^26–28^, the premotor cortex^18,19,29^, the anterior cingulate cortex^17,26,27,30^, left inferior frontal cortex^16,18–20,29,31^, middle frontal gyrus^17^, as well as the insula^17,19,26^. However, the influence of the increased listening effort that hearing-impaired people experience in daily life on long-term changes in resting state functional connectivity is still unknown.

Resting state functional connectivity is described as spontaneous activity that is organized into coherent networks^32^. Several resting state networks, such as the default mode, the dorsal attention and the salience network, have been identified and changes have been investigated in a variety of disorders. The default mode network includes brain regions in frontal, parietal and cingulate cortices that are typically active during rest but inactivated during tasks requiring attention^33–35^. Previous research shows that the activity within the default mode network is decreased in older participants possibly reflecting a deficit in modulating brain activity and resource allocation^34,36–39^. The dorsal attention network involves the frontal eye fields and the intraparietal sulcus and displays an opposite pattern to the default mode network: inactivated during rest but activated during task^35^. The salience network comprises areas in the cingulate cortex, insula, prefrontal and supramarginal cortex and has been related to the detection of salient events, the control of behavior and switching of attention^34,40^. Previous studies show that the salience network is particularly influenced by aging and hearing impairment^1,34,41^.

Few studies investigated resting state connectivity changes in patients with hearing loss and findings are heterogeneous. In mild to moderately hearing-impaired listeners decreased connectivity was found between the dorsal attention network, the insula and postcentral gyrus, as well as increased connectivity between the default mode network and middle frontal gyrus^32^. In contrast, another study showed decreased default mode network but increased dorsal attention network connectivity in hearing-impaired compared to normal-hearing subjects^35^. A recent study by Chen and colleagues demonstrated decreased spontaneous resting state activity in superior temporal gyrus, parahippocampal gyrus and inferior parietal lobule as well as increased activity in middle frontal gyrus, cuneus and postcentral gyrus in hearing-impaired individuals^42^. These alterations were associated with cognitive and language processing deficits. Puschmann and Thiel^43^ investigated whether cross-modal reorganization, which is often observed in deaf^44^ but also mild to moderate hearing-impaired participants^16,45^, impacts functional connectivity and found a changed functional connectivity between visual and auditory cortex during audio-visual processing, as well as during rest.

Differences in brain structure and function in the aging brain can influence sensory and cognitive processing, leading to the assumption of a complex interplay between neuronal structure, function and behavior^38^. How hearing loss and the increased effort that hearing-impaired individuals are exposed to in daily listening situations adds to this complex mechanism is still an unresolved issue. We here aimed to address how uncompensated age-associated hearing loss and the increased listening effort impact whole brain functional connectivity. For that aim, we conducted resting state connectivity MRI in elderly normal-hearing and hearing-impaired participants. We hypothesized that hearing-impaired participants show increased functional connectivity between auditory and visual cortex and altered connectivity in the default mode network, dorsal attention and salience network^32,42,43^. We further expected a relation between increased listening effort and altered functional connectivity in brain regions previously linked to effortful listening in task based fMRI studies such as frontal and premotor cortices, as well as insula and cingulate cortex^16–20,26–28,30^.

## Methods

### Participants

**Figure 1:**
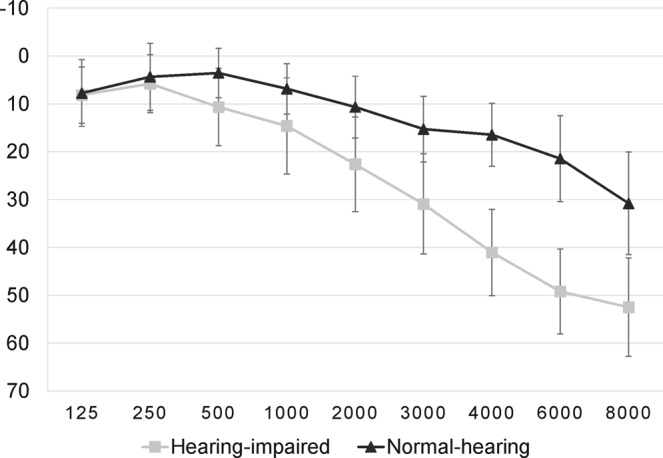
Average pure tone audiograms for hearing-impaired and normal-hearing subjects averaged over both ears. Error bars denote standard deviation.

Thirty-nine subjects participated in the study; one subject was excluded due to movement during the resting state measurement. Nineteen participants were classified as normal-hearing (mean age of 63.2 ± 5 years) and served as control group. The other nineteen participants (mean age of 63.5 ± 5.3 years) were classified as hearing-impaired. Hearing abilities for all participants were 30 dB HL or better for frequencies between 125–1000 Hz. High-frequency hearing loss was defined by more than 30 dB HL for the frequencies 2 to 8 kHz. Further, the hearing loss should be symmetrical. People with values less than 30 dB HL in these frequencies were classified as normal-hearing (cf. WHO, 2001 definition of hearing loss^46^). None of the participants reported current or previous use of a hearing aid. Average pure tone audiograms over both ears for the two groups are depicted in Fig. 1. All participants were right-handed. Exclusion criteria were previous or current psychiatric or neurological disorders. The study was approved by the ethics committee of the University of Oldenburg “Kommission für Forschungsfolgenabschätzung und Ethik” (Committee for research outcome assessment and ethics) and carried out in accordance with the Declaration of Helsinki. All subjects signed a written informed consent form and were paid for participation.

### Experimental procedure

The study consisted of two MRI measurements separated by a short break outside the MRI. The first run was a task based fMRI measurement (results reported in^23^). The second run consisted of a resting state MRI, anatomical MRI and a diffusion MRI measurement. Only the results of the resting state MRI are reported here. Prior to the second run, the participants filled out a listening effort questionnaire (“Höranstrengungsbogen”)^47^ and a handedness inventory^48^. The listening effort questionnaire includes seventeen questions about listening situations in everyday life, e.g. ‘You meet with some friends in a café and are able to see them during the conversation. How effortful is it to follow the conversation?’. Participants are asked to rate the effort they need in the different situations from zero (not effortful at all) to ten (extremely effortful). The mean values over all questions was used as a marker of listening effort. After the MRI, a working memory task (Size Comparison Span)^49^, a pure tone audiometry of the frequencies 125, 250, 500, 1000, 2000, 4000, 6000 and 8000 Hz and a task measuring audiovisual integration (results reported in^23^) were performed.

### Data acquisition

MRI data were acquired by a 3 T whole-body Siemens Magnetom Prisma MRI machine with a 20-channel head coil. Resting state data were recorded from all participants while fixating a white dot presented centrally on a black screen (320 T_2_*-weighted gradient echo planar imaging (EPI) volumes, TR = 1500 ms, TE = 30 ms, voxelsize = 2.2 × 2.2 × 3.0 mm, 25 slices). Structural images were acquired with a 3-D T1-weighted sequence (MP-RAGE, TR = 2300, TE = 4.16, slice thickness 1 mm, 176 sagittal slices).

### Data analysis

We analyzed resting state data with the Statistical Parametric Mapping software package (SPM12, Wellcome Department of Imaging Neuroscience, London, UK) based on Matlab 2016b and the CONN toolbox for SPM^50^. Preprocessing in SPM included realignment estimation, slice timing offset correction, normalization to the Montreal Neurological Institute (MNI) stereotactic space using normalization parameters obtained from a segmentation of the anatomical T1-weighted image and spatial smoothing (full width half maximum = 8 mm). The first five images of the resting state scan were skipped. In the CONN toolbox data processing first included linear regression to remove motion and the BOLD signal from white matter and cerebrospinal fluid. Next linear detrending and bandpass-filtering (0.008–0.09 Hz) was applied. Each subject’s seed-to-voxel connectivity maps of a specific seed to the whole brain (Fisher-transformed correlation coefficients) were entered into a second-level analysis. On the group level, between-subject comparisons and a multiple regression analysis with listening effort values across both groups were performed. In the regression analysis, mean high-frequency hearing thresholds (2 to 8 kHz) were used as covariates to identify brain regions related to subjective listening effort independent of the severity of hearing loss. Regions of interest included the auditory cortex as well as the default mode network, salience network and dorsal attention network (see seed regions including MNI coordinates in Table 1, implemented in the CONN toolbox). For the networks, averages over all seeds were used in the analysis. The seed in the auditory cortex (MNI coordinates: −56, −10, 2) was selected based on its highest peak in the task-based part of the study (taken from^23^). For all data, effects were determined to be significant when passing a threshold of p < 0.05 (FWE cluster size inference). Bonferroni-correction was applied to correct for multiple comparisons (i.e. three networks). Peak coordinates are reported in MNI space. All measures throughout the text and in Fig. 1 refer to mean values (±standard deviation).

**Table 1:** Seed regions for different networks used in the resting state analysis.

Network	Brain region	MNI coordinates
Default Mode	Medial prefrontal cortex	(1, 55, −3)
	Parietal lobe (left)	(−39, −77, 33)
	Parietal lobe (right)	(47, −67, 29)
	Posterior cingulate (right)	(1, −61, 38)
Salience	Anterior cingulate	(0, 22, 35)
	Anterior Insula (left)	(−44, 13, 1)
	Anterior Insula (right)	(47, 14, 0)
	Prefrontal cortex (left)	(−32, 45, 27)
	Prefrontal cortex (right)	(32, 46, 27)
	Supramarginal gyrus (left)	(−60, −39, 31)
	Supramarginal gyrus (right)	(62, −35, 32)
Dorsal Attention	Frontal eye field (left)	(−27, −9, 64)
	Frontal eye field (right)	(30, −6, 64)
	Intraparietal sulcus (left)	(−39, −43, 52)
	Intraparietal sulcus (right)	(39, −42, 54)

## Results

### Cognition

Working memory scores were 11.47 (±12.8) for the hearing-impaired group and 12.47 (±9) for the normal-hearing group. From participation in other studies we gathered data for 11 hearing-impaired and 13 normal-hearing participants measuring cognitive decline^51^ with mean values of 25.8 for hearing-impaired and 26.9 for normal-hearing participants. All measures refer to normal cognitive functioning. Significant differences between both groups were not obtained.

### Hearing loss and listening effort

Mean values of hearing loss for the high frequencies (2 to 8 kHz) were 39.16 ± 6.1 dB for the hearing-impaired group and 18.92 ± 6.52 dB for the normal-hearing group. Mean values for the low frequencies (125 to 1000 Hz) were 9.8 ± 6.82 dB for the hearing-impaired group and 5.63 ± 5.16 dB for the normal-hearing group. Listening effort was significantly higher in the hearing-impaired group (3.27 ± 1.65) than in the normal-hearing participants (2.26 ± 1.19) (T(36) = 2.128; p = 0.02). High-frequency hearing loss and listening effort correlated significantly (r = 0.379; p = 0.019).

### Resting state data

#### Functional connectivity in hearing-impaired and normal-hearing participants

**Figure 2:**
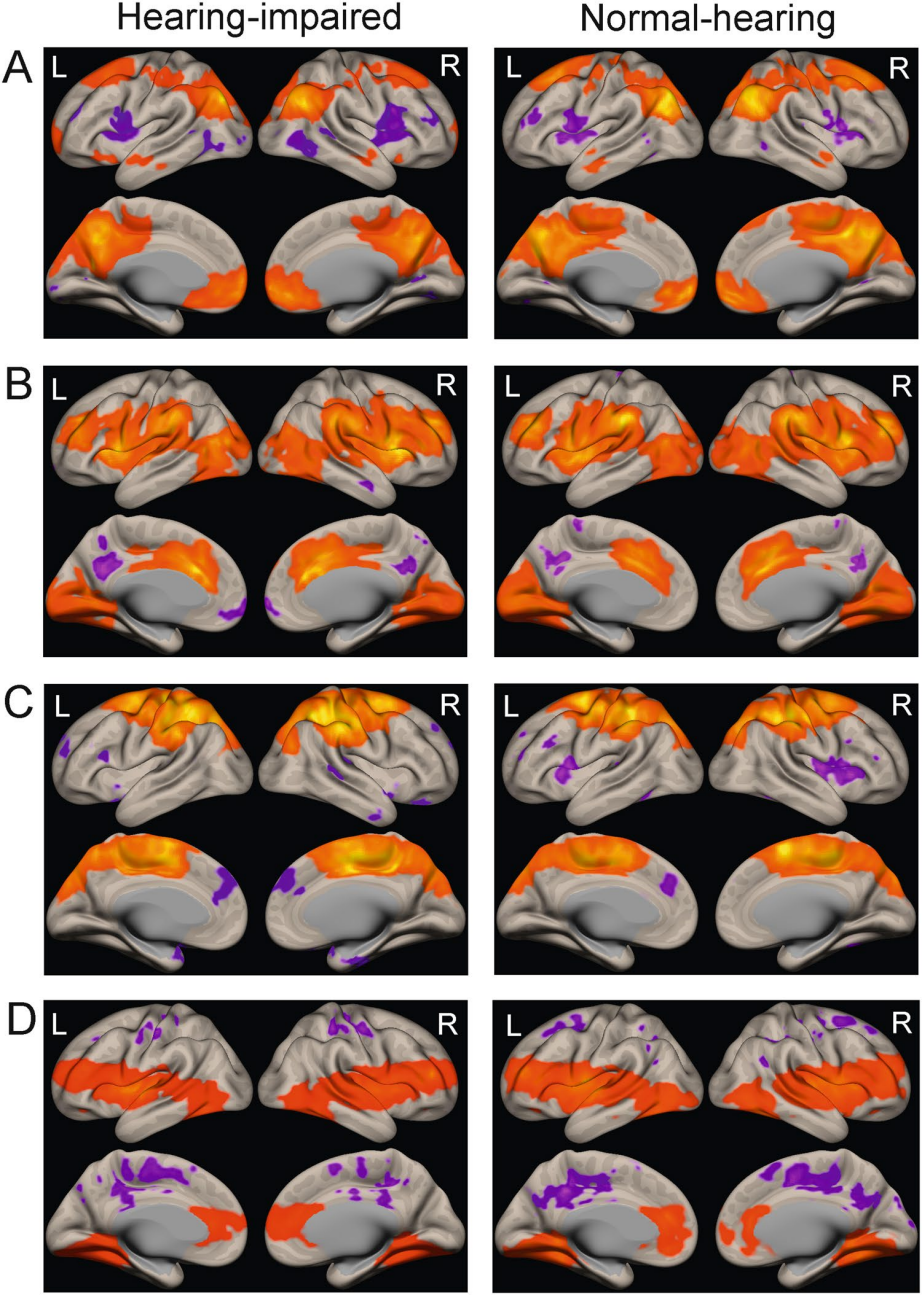
Brain regions that are functionally coupled with the (**A**) Default mode network, (**B**) Salience network, (**C**) Dorsal Attention network, (**D**) Auditory cortex in hearing-impaired (left) and normal-hearing participants (right). Brain regions depicted in red/orange are positively coupled with the respective network, brain regions in blue/purple are negatively coupled (i.e. anticorrelated) with the respective network; L and R denote left and right hemispheres respectively [p < 0.05; FWE corrected on the cluster level].

Average connectivity patterns of seed regions for each resting state network can be seen in Fig. 2 for normal-hearing and hearing-impaired subjects. No significant differences between hearing-impaired and normal-hearing participants were found. A multiple regression analysis with high-frequency hearing loss did also not reveal any significant changes in functional connectivity as a function of hearing loss.

#### The effect of listening effort on functional connectivity

**Figure 3:**
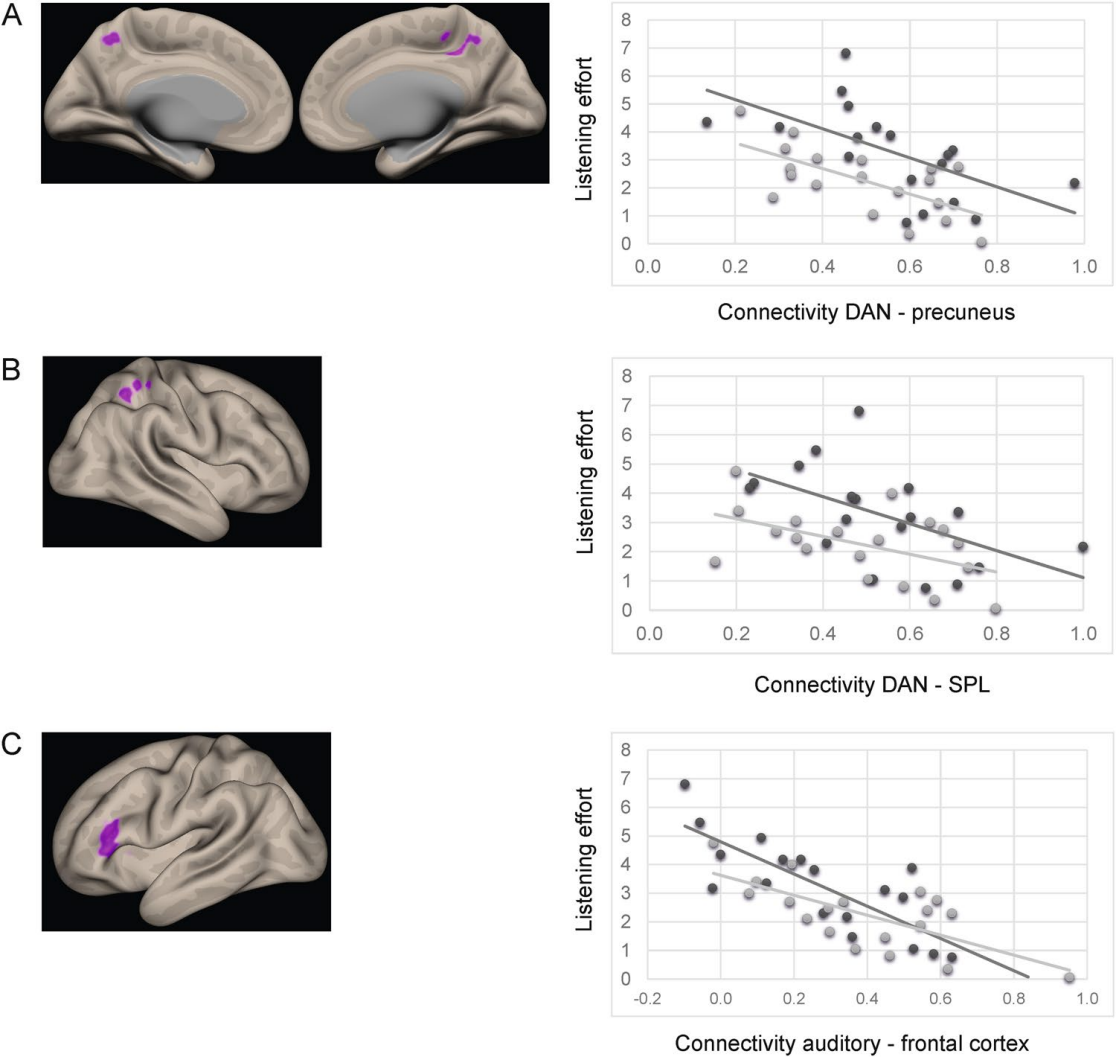
Correlation between listening effort and functional connectivity of (**A**) dorsal attention network (DAN) to precuneus, (**B**) dorsal attention network (DAN) to superior parietal lobule (SPL) and (**C**) auditory seed to inferior frontal gyrus. Left side displays inflated brain views and right side shows the correlation between connectivity and listening effort for hearing-impaired (dark dots) and normal-hearing participants (bright dots); correlations between groups are not significantly different.

Resting state connectivity varied as a function of listening effort. There was a significant negative correlation between connectivity in the dorsal attention network to the precuneus and to superior parietal lobule (Table 2 and Fig. 3A,B). Further, an increased listening effort was found to be negatively correlated with connectivity between the auditory cortex and inferior frontal gyrus (Table 2 and Fig. 3C). In other words, an increase in the experienced daily listening effort was related to lower functional connectivity of the dorsal attention network and auditory cortex. For completeness we also report two correlations which did not survive the Bonferroni-correction for multiple comparisons: (1) a negative correlation between listening effort and connectivity between default mode network and postcentral gyrus (MNI-coordinate: 30, −26, 42; z-value: 4.82) and (2) and a positive correlation between listening effort and connectivity between auditory seed to precentral gyrus (MNI-coordinate: 12, −30, 60; z-value: 4.67).

**Table 2:** Peak MNI coordinates for multiple regression with listening effort and covariate hearing loss.

Seed region	Peak coordinates (x, y, z)	Z-score	Cluster size	Brain region
Dorsal attention network	(4, −40, 48)	4.05	260	Precuneus
	(26, −48, 44)	3.99	240	Superior parietal lobule
Auditory cortex	(−50, 32, 14)	5.54	274	Inferior frontal gyrus

## Discussion

We investigated functional resting state connectivity in normal-hearing and mild to moderate hearing-impaired elderly listeners and its relation to listening effort. We did not find any differences in functional connectivity between normal-hearing and hearing-impaired listeners, neither for any of the assessed networks nor for the auditory cortex. Listening effort, however, was related to reduced resting state connectivity between the dorsal attention network and the precuneus as well as the superior parietal lobule. Further, increased listening effort was associated with a decreased connectivity between auditory and inferior frontal cortex.

### Functional connectivity in in age-related hearing loss

Differences in resting state connectivity between hearing-impaired and normal-hearing participants were not obtained. In contrast, Husain and colleagues^32^ showed decreased connectivity between seed regions in the dorsal attention network with the insula and postcentral gyrus, and increased connectivity between seeds in the default mode network and middle frontal gyrus in mild to moderate hearing-impaired participants. In a prior study, Schmidt and colleagues^35^ compared tinnitus patients with hearing-impaired participants and normal-hearing participants. Although not focus of the study, they found an increased connectivity of the dorsal attention network to the insula in hearing-impaired participants as compared to normal-hearing subjects^35^. Recently, Chen *et al*.^42^ showed a decreased connectivity in superior temporal gyrus (BA 38), precuneus and inferior parietal lobule as well as increased connectivity in middle frontal gyrus, cuneus and postcentral gyrus in hearing-impaired compared to normal-hearing participants. One explanation for the lack of significant differences between hearing-impaired and normal-hearing participants in our study could be the relatively mild to moderate hearing impairment of the participants. However, the impairment in two of the other studies cited above^32,35^ was also mild to moderate with slightly younger subjects. Their normal-hearing participants had a mean age of 50 years while ours had a mean age of 63 years (matched to the hearing-impaired group) with age appropriate hearing abilities. Hence, it may also be the case that differences in resting state connectivity that are present in middle-aged hearing-impaired subjects may be covered by larger age-associated changes in connectivity with increasing age. Other research showed that age has a significant effect on resting state networks^34,37–39,41^.

Concerning the direct coupling of visual and auditory cortex, Husain and colleagues^32^ did also not find any connectivity differences with seeds in the auditory cortex between hearing-impaired and normal-hearing participants and relate these findings to the mild to moderate severity of hearing loss in their study. Chen *et al*.^42^ did also not find any significant effects in primary auditory cortex. On the other hand, a prior study in our lab^43^ did find an increased connectivity between auditory and visual cortex as a function of hearing loss. Since all of these studies–including ours–employed an eyes-open and fixation cross paradigm for the resting state measurement, the paradigm itself could not be the underlying cause of different results. In contrast to the prior study where the resting state measurement directly followed an audiovisual task, participants in the current study had a short break outside the MRI directly before the resting state measurement. Hence, it is not clear whether take-over effects of previous tasks may have contributed to the different results^52^.

### The effect of listening effort on functional connectivity

Apart from hearing abilities determined by the audiogram we assessed subjective listening effort in everyday situations. Our results suggest that an increase in individually perceived everyday listening effort is associated with a decrease in resting state coupling between the dorsal attention network and the precuneus and superior parietal lobule as well as between the auditory and the inferior frontal cortex.

There is some evidence of resting state connectivity changes in the dorsal attention and default mode network in age-related hearing loss^32^. We here suggest that listening effort experienced in daily life may be one important factor for changed functional connectivity between the dorsal attention network and the precuneus and superior parietal lobule. The precuneus is part of the default mode network and plays a role in several diseases, including Alzheimer’s^33,36,37,53–55^ but also tinnitus. Tinnitus patients exhibit decreased connectivity between the precuneus and other regions of the default mode network, speaking for a less coherent network due to the phantom sound^35^. Chen and colleagues^42^ demonstrated neuronal changes in superior temporal gyrus, prefrontal cortex, precuneus and inferior parietal lobule as part of the default mode network in age-related hearing loss. They suggest that decreased connectivity values in the precuneus are responsible for disrupting the default mode network in age-related hearing loss. We here show a disruption of coupling between dorsal attention and default mode network (via precuneus). Dorsal attention and default mode network are often anti-correlated, the dorsal attention network being active during tasks and the default mode network being active at rest. Disruptions in this coupling can be associated with decreased cognitive functions^56^ or aging^34,36,37,39^. In long-term tinnitus patients, there was also a decreased connectivity between the dorsal attention network and precuneus^57^. The decreased connectivity is explained as a suppression and habituation mechanism in response to the phantom sound. Although we excluded participants experiencing tinnitus in our study, the connectivity between the dorsal attention network and precuneus was decreased with high listening effort. In age-related untreated hearing loss, the increased listening effort experienced in daily life may therefore result in a similar disruption between dorsal attention and default mode network as in tinnitus patients. Decreases in functional connectivity were also found within the dorsal attention network, i.e. to the superior parietal lobule. Hence, our data suggest a disruption of between and within network connectivity of the dorsal attention network with increased listening effort.

Moreover, listening effort was related to a decreased connectivity between auditory and inferior frontal cortex. These results are in line with previous work in hearing-impaired participants showing increased frontal recruitment as a compensatory mechanism to control for the decreased auditory input^16,17,21,22,45^. An increase in frontal activity was previously also associated with effortful listening in healthy participants^19,20,26–31^. Moreover, effortful speech perception was related to an increase in neural activation in lateral temporal cortex, inferior frontal cortex and premotor cortex^18^. The inferior frontal gyrus belongs to the core speech network involved in processing acoustic, phonological and lexico-semantic and basic syntactic material^29,31,58^. Additonally, the inferior frontal cortex is connected to the auditory cortex and serves as top-down modulatory control to faciliatate speech understanding. Connections between auditory regions to prefrontal areas, including inferior frontal cortex, are thought to function as a modulatory control and compensatory mechanism during effortful listening^18^. Previous research showed that speech understanding in adverse listening situations is facilitated by top-down influences accomplished via inferior frontal and premotor cortex, as well as superior temporal sulcus and parietal cortex^29,31^. Hence, there is compelling evidence that the inferior frontal cortex has an important role in speech understanding and is particularly involved in processing speech under difficult listening situations. Our results add further evidence, since we show a connection of everyday listening effort and a disruption in resting state coupling between auditory and inferior frontal cortex. Participants experiencing high everyday life listening effort showed a decreased connectivity between auditory cortex and inferior frontal cortex.

There are two possible explanations for this decreased coupling. First, it may be possible that people experiencing high listening effort have a general coupling deficiency between auditory and frontal cortex which may impact both, the coupling during effortful speech perception and during rest. This may also lead to decreased performance in speech understanding. The second suggestion to explain our results might be that individuals experiencing high listening effort present a higher connectivity between auditory and frontal cortex during effortful listening tasks which may be followed by reduced coupling in situations where the effort is no longer needed. The latter suggestion of increased connectivity during task and decreased connectivity during rest can be better reconciled with the many fMRI studies demonstrating that increased involvement of the frontal lobe during effortful speech perception can facilitate speech understanding. Additional evidence comes from our own previous study in the same sample of subjects, which showed an increased frontal recruitment and preserved performance in speech understanding in hearing-impaired participants^23^. Future studies examining both functional connectivity during speech understanding and during resting state are needed to entangle the underlying processes related to the increased listening effort, changes in functional connectivity and the resulting impact on speech understanding.

## Conclusion

The variety of resting state connectivity changes related to listening effort seem to reflect more widespread changes in underlying network dynamics. Listening effort experienced in everyday life rather than the hearing loss itself has a significant influence on resting state connectivity. The underlying nature for these changes seems to be a compensatory mechanism to control for decreased auditory input, increased listening effort and decreased neural resources. With regard to the scarce research dealing with functional connectivity changes in mild to moderate age-related hearing loss, we provide new evidence for plastic changes not only in auditory cortex but also in coupling between dorsal attention network, precuneus and superior parietal lobule affecting different resting state networks. We conclude that mainly the individually perceived listening effort in elderly participants leads to altered coupling between different brain areas independent of the hearing impairment itself.
